# Moderate and High Disease Activity Predicts the Development of Carotid Plaque in Rheumatoid Arthritis Patients without Classic Cardiovascular Risk Factors: Six Years Follow-Up Study

**DOI:** 10.3390/jcm10214975

**Published:** 2021-10-27

**Authors:** Iván Ferraz-Amaro, Alfonso Corrales, Belén Atienza-Mateo, Nuria Vegas-Revenga, Diana Prieto-Peña, Ricardo Blanco, Miguel Á. González-Gay

**Affiliations:** 1 Division of Rheumatology, Hospital Universitario de Canarias, 38320 Tenerife, Spain; iferrazamaro@hotmail.com; 2 Epidemiology, Genetics and Atherosclerosis Research Group on Systemic Inflammatory Diseases, Hospital Universitario Marqués de Valdecilla, IDIVAL, 39011 Santander, Spain; afcorralesm@hotmail.com; 3 Division of Rheumatology, Hospital Universitario Marqués de Valdecilla, Universidad de Cantabria, 39008 Santander, Spain; mateoatienzabelen@gmail.com (B.A.-M.); nuriavegas2@gmail.com (N.V.-R.); diana.prieto.pena@gmail.com (D.P.-P.); rblancovela@gmail.com (R.B.); 4 Cardiovascular Pathophysiology and Genomics Research Unit, Faculty of Health Sciences, School of Physiology, University of the Witwatersrand, Johannesburg 2000, South Africa

**Keywords:** rheumatoid arthritis, carotid plaque, prospective study

## Abstract

Patients with rheumatoid arthritis (RA) have a higher incidence of subclinical atherosclerosis and cardiovascular (CV) disease. It is postulated that the appearance of accelerated atherosclerosis in these patients is a consequence of the inflammation present in the disease. In this study, we aim to determine if baseline disease activity in patients with RA predicts the future development of carotid plaque. A set of consecutive RA patients without a history of CV events, cancer or chronic kidney disease, who did not show carotid plaque in a carotid ultrasound assessment, were prospectively followed up for at least 5 years. At the time of recruitment, CV risk factors and disease-related data, including disease activity scores, were assessed. At the end of the follow-up, a carotid ultrasound was repeated and patients were divided into two groups; those who developed carotid plaque, and those who did not. A multivariable regression analysis was performed to define the predictors for the development of carotid plaque. One hundred and sixty patients with RA were followed up for an average of 6 ± 1 years. After this time, 66 (41%) of the patients had developed carotid plaque, and 94 (59%) did not. Patients with carotid plaque were significantly older (47 ± 13 vs. 55 ± 9 years, *p* < 0.001) at baseline, were more frequently diabetic (0% vs. 6%, *p* = 0.028), and had higher total cholesterol (197 ± 36 vs. 214 ± 40 mg/dL, *p* = 0.004) and LDL cholesterol (114 ± 35 vs. 126 ± 35 mg/dL, *p* = 0.037) at the beginning of the study. After multivariable adjustment, patients who were in the moderate and high disease activity (DAS28-CRP) categories displayed a higher odds ratio for the appearance of carotid plaque (OR 2.26 [95% CI 1.02–5.00], *p* = 0.044) compared to those in the DAS-28-CRP remission category. Remarkably, when patients were divided in patients within the low-risk SCORE category, and patients included in the remaining SCORE categories (moderate, high and very high), the relation between DAS28-CRP and the development of carotid plaque was only significant in the low-risk SCORE category. In conclusion, disease activity predicts the future development of subclinical atherosclerosis in patients with RA.

## 1. Introduction

Rheumatoid arthritis (RA) has been associated with a higher incidence and prevalence of cardiovascular (CV) events [1] and CV mortality [2]. This increased risk of CV disease does not appear to be fully mediated by traditional CV risk factors for atherosclerosis [3]. It is known that in addition to the traditional CV risk factors and a genetic component [4], the presence of chronic inflammation can explain the development of accelerated atherosclerosis in these patients [5] through effects mediated by cytokines, immune complexes and endothelial dysfunction, or by a combination of these factors [4,6].

CV disease risk prediction algorithms, which were originally developed for use in the general population and relied heavily on traditional CV risk factors, have been found to have suboptimal performance in patients with RA [7]. For this reason, it is of great interest to implement surrogate markers that allow us to identify patients with RA at high risk of developing CVD events. In this regard, a recent study of patients with RA followed prospectively has shown that the presence of carotid plaques assessed by carotid ultrasound predicts the risk of CV events [8]. Accordingly, the presence of carotid plaques identifies a subgroup of patients with RA at high risk of CV events.

Interestingly, certain treatments for RA, in particular the use of biologic therapy, prevent the development of CV disease [9]. These findings suggest that reducing the inflammatory burden and inducing remission of the disease may have a favorable effect on the CV risk of patients with RA. However, there are no long-term prospective studies that directly link disease activity with the subsequent development of CV disease.

Taking these considerations into account, in the present work, we have prospectively followed up a group of patients with RA in whom the presence of carotid plaque was ruled out at the beginning of the study. After at least five years, we set out to establish what the predictors of carotid plaque development were in those RA patients who developed carotid plaques.

## 2. Materials and Methods

### 2.1. Study Participants

This study included a series of patients with RA in whom the presence of carotid plaque was excluded by carotid ultrasound. All were 18 years or older, and were included in the study if they met the 2010 ACR/EULAR classification criteria for RA [10]. After at least 5 years, a carotid ultrasound was performed again. Patients were divided into those who developed carotid plaque and those who did not.

For the purpose of inclusion in the present study, RA disease duration at the beginning of the study needed to be ≥1 year. Patients taking glucocorticoids were included only if they were taking an equivalent dose of ≤10 mg/day of prednisone. However, patients were excluded if they had a history of previous CV events, cancer or any other chronic disease, evidence of active infection or a glomerular filtration rate < 60 mL/min/1.73 m^2^. Patients who did not undergo the 5-year carotid ultrasound evaluation for any reason (such as, they had experienced CV events, died, or moved to another region during follow-up) were not included in the evaluation. The study protocol was approved by the Institutional Review Committees at Hospital Marqués de Valdecilla, Spain (Approval reference: 17/2012). All subjects provided informed written consent.

### 2.2. Data Collection and Laboratory Assessments

At the beginning of the study, patients completed a CV risk factor assessment, a medication use questionnaire, and underwent a physical examination at baseline. Weight, height, body-mass index, abdominal circumference, and systolic and diastolic blood pressure were assessed under standardized conditions. Information regarding smoking status and hypertension was obtained from the questionnaire at baseline. Medical records were reviewed to ascertain specific diagnoses and medications. Hypertension was defined as systolic or diastolic blood pressure higher than 140 and 90 mmHg, respectively. Cholesterol, triglycerides, and HDL cholesterol were measured using an enzymatic colorimetric assay. LDL cholesterol was calculated using the Friedewald formula. The standard technique was used to measure the erythrocyte sedimentation rate (ESR) and high-sensitivity C-reactive protein (CRP). Disease activity in patients with RA was measured using the Disease Activity Score (DAS28-CRP) in 28 joints [11]. As described elsewhere [12], DAS28-CRP categories were defined as <2.6: remission; ≥2.6 and ≤3.2: low disease activity; >3.2 and ≤5.1: moderate disease activity, and >5.1: high disease activity.

### 2.3. Carotid Ultrasound Assessment

A carotid ultrasound examination, at baseline and after 5 years of follow-up, was performed to assess carotid intima-media wall thickness (cIMT) in the common carotid artery and to detect focal plaques in the extracranial carotid tree in patients with RA [13]. A commercially available scanner was used for this purpose in real-time: the Esaote Mylab 70 (Genoa, Italy), equipped with a 7–12 MHz linear transducer and an automated software-guided radiofrequency technique; quality intima-media thickness (QIMT, Esaote, Maastricht, The Netherlands). As previously reported [14], based on the Mannheim consensus, plaque criteria in the accessible extracranial carotid tree (common carotid artery, bulb and internal carotid artery) were defined as follows: a focal protrusion in the lumen measuring at least cIMT > 1.5 mm; a protrusion at least 50% greater than the surrounding cIMT; or, arterial lumen encroaching > 0.5 mm.

### 2.4. Statistical Analysis

Demographic and clinical characteristics are shown as frequencies for binary variables. Continuous variables were expressed as mean ± standard deviation (SD), or median and interquartile range (IQR) for non-normally distributed variables. Univariable differences between patients who developed carotid plaque and those that did not at 5 years of follow-up were evaluated using the Student’s *t*, Mann–Whitney U, chi-square or Fisher’s exact tests, according to the normal distribution and the number of subjects. Baseline characteristics associated with the development of carotid plaque were calculated through logistic multivariable regression analysis. Odds ratios (OR) were adjusted for those variables with a *p*-value inferior to 0.10 in the univariable difference between patients with carotid plaque and without carotid plaque. All the analyses used a 5% two-sided significance level and were performed using SPSS software, version 26 (IBM Corp, Armonk, NY, USA). A *p*-value < 0.05 was considered statistically significant.

## 3. Results

### 3.1. Demographics, Cardiovascular Risk Factors, and Disease-Related Data in RA Patients at Baseline

One hundred and sixty patients with RA were followed up for at least 5 years. The baseline age of these patients was 50 ± 12 years, and 83% of the patients were women. Regarding traditional CV risk factors observed at the baseline, 27% of the patients were current smokers, 29% had obesity (a body mass index equal to or greater than 30 kg/m^2^), and 28% and 3% of them had, respectively, hypertension and diabetes (Table 1). Lipid profile values at baseline are shown in Table 1. Additionally, 13% of the patients were taking statins, 19% were undergoing antihypertensive treatment and 2% were taking aspirin. The mean cIMT was 620 ± 108 microns, and as required for inclusion in the study, none of the patients had carotid plaque at baseline.

Regarding disease-related data, the baseline median disease duration was 7 (IQR 4–12) years, and at least half of the patients were positive for rheumatoid factor (58%) or anti-citrullinated protein antibodies [ACPAs] (53%). The ESR was 11 (IQR 5–7) mm/1st hour, and the CRP value was 1.9 (IQR 0.5–4.0) mg/L. The average DAS28-CRP was 2.78 ± 1.10, including most of the patients in the remission category (45%). Furthermore, 21% were in the low disease activity category, and 34% were in the moderate or high disease activity categories. Regarding RA therapies, 44% of the patients were taking prednisone at a median dose of 5 (IQR 2.5–7.5) mg/day, and 63% and 35% were taking methotrexate and biologic therapies, respectively (Table 1).

### 3.2. Predictors of Development of Carotid Plaque after Six Years of Follow-Up

After at least 5 years of follow up, 66 (41%) of the patients had developed carotid plaque. Remarkably, DAS28-CRP decreased during follow-up from 2.78 ± 1.10 at baseline to 2.62 ± 1.23 by the end of the study (mean difference 0.16 ± 1.25, *p* = 0.11). Table 2 shows the baseline differences between patients with carotid plaque and those without carotid plaque at the end of the study. The average time of follow-up was 6 ± 1 years in both groups. Patients who developed carotid plaque were significantly older (55 ± 9 vs. 47 ± 13 years, *p* < 0.001) at baseline; were more frequently diabetic (6% vs. 0%, *p* = 0.028); and had a higher total cholesterol (214 ± 40 vs. 197 ± 36 mg/dL, *p* = 0.004) and LDL-cholesterol (126 ± 35 vs. 114 ± 35 mg/dL, *p* = 0.037) at the beginning of the study. As expected, patients who developed carotid plaque at the end of the follow-up had a higher cIMT at baseline than those who did not (655 ± 103 vs. 595 ± 109 microns, *p* < 0.001) (Table 2).

Regarding disease-related data, RA duration and rheumatoid factor or ACPAs were not associated with the development of carotid plaque over time. In contrast, baseline disease activity was found to predict the development of carotid plaque. Therefore, DAS28-CRP, when considered continuously, was associated with an almost significantly higher probability of developing carotid plaque (OR 1.38 [95% CI 1.00–1.02], *p* = 0.052). When this analysis was performed using DAS28-CRP categories as an ordinal variable, patients who had moderate or high disease activity at baseline were at higher risk of developing carotid plaque, compared with those who were in the remission category (OR 2.26 [95% CI 1.02–5.00], *p* = 0.044) (Table 2). This association was found after a multivariable analysis adjustment was made for traditional CV risk factors.

### 3.3. Relationship between Disease Activity and the Development of Carotid Plaque in Different SCORE CV Risk Categories

We additionally performed an analysis of the relationship between disease activity and the development of carotid plaque separately in two groups of patients: subjects within the low-risk SCORE category, and patients included in the remaining SCORE categories (moderate, high and very high). Remarkably, the association of DAS28-CRP with carotid plaque was only significant in the low-risk SCORE category. In this regard, being in the low disease activity vs. remission category displayed an OR of 5.24 (95% CI 1.16–23.54), *p* = 0.031, and belonging to the moderate to high disease activity vs. remission showed an OR of 4.38 (95% CI 1.17–16.38), *p* = 0.028, for the development of carotid plaque (Table 3). The same analysis was not significant in the subgroup of patients included in the moderate to high and very high-risk SCORE categories (Table 3).

## 4. Discussion

Although evidence of a relationship between RA and CV disease is extensive, the literature lacks prospective studies on this issue. According to our findings, patients with high disease activity at the start of prospective follow-up will double the risk of subclinical atherosclerosis after 6 years compared with those in remission. This is relevant, as it was found after adjusting for classic CV risk factors.

In a previous report of 24,989 patients followed for a median of 2.7 years, a 10-point reduction in the time-averaged clinical disease activity index was found to be associated with a 26% reduction in CV risk [15]. The authors of this study concluded that a reduction in time-averaged disease activity in RA would lead to fewer CV events. A former study of the same group discovered baseline RA characteristics were found to independently predict future CV events in a RA cohort followed for a median of 22 months [16]. In this report, a CV event prediction model was significantly improved when RA severity markers were added to the same model that contained only classic CV risk factors. Similarly, in another report based on prospectively collected data, time-averaged DAS28 was significantly associated with CV disease after correction for confounders [17]. Unlike previous studies, our work used the presence of carotid plaque as an endpoint. It should be noted that the follow-up of our series was considerably longer, reaching up to 6 years. Despite these differences, our work remains consistent with the aforementioned studies.

The presence of carotid plaques has been shown to predict the development of CV events and death in patients with RA [8]. This was demonstrated in a previous study that included 327 RA patients followed for 5 years. Since carotid plaque predicts CV events and mortality, and disease activity also predicts carotid plaque development, we can assume that disease activity may lead to increased subclinical atherosclerosis that will eventually produce an increased incidence of CV events in RA patients who have high disease activity.

In line with the above, in a previous study, it was shown that the magnitude and chronicity of the inflammatory response measured by longitudinal evaluation of CRP levels, rather than by a single determination of this inflammatory marker, correlated directly with cIMT [5]. In our present study, baseline serum CRP levels by themselves had no predictive capacity for the development of carotid plaque. However, the DAS28-CRP activity score did have this ability. We believe this is because the score includes other variables that have to do with inflammation, such as the presence of tender and swollen joints.

In our work, the association between disease activity and the development of carotid plaque was found in patients within the low-risk SCORE category, but not in patients who were in other SCORE risk categories. Given that CV disease in RA has been attributed to classic (traditional) and non-traditional CV risk factors, we believe that patients who have fewer classic CV risk factors are those who are more predisposed to express the influence of the activity of disease over the CV disease.

In conclusion, our study reinforces the claim that accelerated atherosclerosis in RA patients may be a consequence of the inflammatory state present in the disease. Controlling disease activity and achieving remission in these patients would likely prevent future development of subclinical atherosclerosis and subsequently CV events in patients with RA.

## Figures and Tables

**Table 1:** Demographics, cardiovascular risk factors, and disease-related data in rheumatoid arthritis patients at baseline.

	Patients (*n* = 160)
Age, years	50 ± 12
Women, *n* (%)	133 (83)
Cardiovascular data	
CV risk factors, *n* (%)	
Current smoker	43 (27)
Obesity	47 (29)
Hypertension	45 (28)
Diabetes Mellitus	4 (3)
BMI, kg/m^2^	27 ± 8
Abdominal circumference, cm	91 ± 24
Lipids	
Total cholesterol, mg/dL	204 ± 38
Triglycerides, mg/dL	100 ± 49
HDL-cholesterol, mg/dL	61 ± 17
LDL-cholesterol, mg/dL	119 ± 35
Atherogenic index	3.5 ± 1.0
Statins, *n* (%)	21 (13)
Hypertension treatment, *n* (%)	30 (19)
Aspirin, *n* (%)	3 (2)
SCORE	0 (0–1.5)
SCORE categories, *n* (%)	
Low risk	90 (56)
Moderate risk	68 (43)
High risk	0 (0)
Very-high risk	2 (1)
Disease-related data	
Disease duration, years	7 (4–12)
CRP, mg/L	1.9 (0.5–4.0)
ESR at time of study, mm/1st hour	11 (5–17)
Rheumatoid factor, *n* (%)	92 (58)
ACPA, *n* (%)	84 (53)
DAS28-PCR	2.78 ± 1.10
Remission	72 (45)
Low disease activity	34 (21)
Moderate and high activity	54 (34)
Treatments, *n* (%)	
Prednisone	70 (44)
Prednisone doses, mg/day	5 (2.5–7.5)
NSAIDs	64 (40)
Methotrexate	100 (63)
Biologic therapy	56 (35)
Subclinical atherosclerosis	
Carotid IMT, microns	620 ± 108
Carotid plaques, *n* (%)	0 (0)

Data represent mean ± SD, or median (IQR) when data were not normally distributed. ACPA: Anti-citrullinated protein antibodies. BMI: Body mass index. CRP: C-reactive protein. CV: Cardiovascular. DAS28: Disease Activity Score in 28 joints. DMARD: Disease-modifying antirheumatic drug. ESR: Erythrocyte sedimentation rate. HDL: High-density lipoprotein. IMT: Intima-media thickness. LDL: Low-density lipoprotein. NSAIDs: Nonsteroidal anti-inflammatory drugs. SCORE: Systematic Coronary Risk Evaluation. SCORE categories: Low (<1%), moderate (1–4%), high (5–9%) or very high (>10%).

**Table 2:** Multivariate analysis of the relationship between baseline characteristics and the development of carotid plaque.

	No Carotid Plaque		Carotid Plaque	OR (95% CI)	*p*
	(*n* = 94)	(*n* = 66)	*p*		
Average follow-up, years	6 ± 1	6 ± 1	0.99		
Age, years	47 ± 13	55 ± 9	**<0.001**		
Women, *n* (%)	81 (86)	52 (79)	0.22		
Cardiovascular data					
CV risk factors, *n* (%)					
Current smoker	21 (22)	22 (33)	0.12		
Obesity	25 (27)	22 (33)	0.36		
Hypertension	28 (30)	17 (26)	0.56		
Diabetes Mellitus	0 (0)	4 (6)	**0.028**		
BMI, kg/m^2^	26 ± 8	28 ± 7	0.14		
Abdominal circumference, cm	87 ± 23	96 ± 25	0.25		
Lipids					
Total cholesterol, mg/dL	197 ± 36	214 ± 40	**0.004**		
Triglycerides, mg/dL	96 ± 48	106 ± 51	0.25		
HDL-cholesterol, mg/dL	60 ± 16	64 ± 18	0.19		
LDL-cholesterol, mg/dL	114 ± 35	126 ± 35	**0.037**		
Atherogenic index	3.49 ± 0.97	3.57 ± 0.99	0.60		
Statins, *n* (%)	13 (14)	8 (12)	0.75		
Hypertension treatment, *n* (%)	15 (16)	15 (23)	0.31		
Aspirin, *n* (%)	1 (1)	2 (3)	0.57		
SCORE	0 (0–1)	0 (1–1.5)	0.084		
SCORE categories, *n* (%)					
Low risk	60 (64)	30 (45)	0.041		
Moderate risk	33 (35)	35 (53)			
High risk	0 (0)	0 (0)			
Very-high risk	1 (1)	1 (2)			
Disease-related data					
Disease duration, years	6 (4–6)	8 (4–8)	0.99		
CRP, mg/L	1.5 (0.5–4.1)	2.0 (0.7–4.0)	0.95		
ESR, mm/1° hour	11 (5–18)	11 (4–16)	0.57		
Rheumatoid factor, *n* (%)	55 (59)	37 (56)	0.76		
ACPA, *n* (%)	47 /50)	37 (56)	0.52		
DAS28-CRP	2.6 ± 1.0	3.0 ± 1.1	**0.015**	1.38 (1.00–1.02)	0.052
Remission	49 (52)	23 (35)	**0.049**	-	-
Low activity	20 (21)	14 (21)		1.35 (0.54–3.38)	0.52
Moderate and high activity	25 (27)	29 (44)		**2.26 (1.02–5.00)**	**0.044**
Treatments, *n* (%)					
Prednisone	40 (43)	30 (45)	0.72		
Prednisone doses, mg/day	2.75 (2.5–2.75)	5 (5–5)	0.087	1.21 (0.95–1.54)	0.12
NSAIDs	33 (35)	31 (47)	0.13		
Methotrexate	61 (65)	39 (59)	0.46		
Biologic therapy	31 (33)	25 (38)	0.52		
Subclinical atherosclerosis					
Carotid IMT, microns	595 ± 109	655 ± 103	**<0.001**		

Data represent mean ± SD or median (IQR) when data were not normally distributed. ACPA: Anti-citrullinated protein antibodies. BMI: Body mass index. CRP: C-reactive protein.CV: Cardiovascular. DAS28: Disease Activity Score in 28 joints. DMARD: Disease-modifying antirheumatic drug. ESR: Erythrocyte sedimentation rate. HDL: High-density lipoprotein. TNF: Tumor necrosis factor. IMT: Intima-media thickness. LDL: Low-density lipoprotein. NSAIDs: Nonsteroidal anti-inflammatory drugs. SCORE: Systematic Coronary Risk Evaluation. SCORE categories: Low (<1%), moderate (1–4%), high (5–9%) or very high (>10%). OR are adjusted for variables with a *p*-value inferior to 0.10 in the univariable analysis. (Age, diabetes mellitus and total cholesterol). LDL-cholesterol was not included in the multivariable analysis because of the collinearity with total cholesterol. Significant *p* values are depicted in bold.

**Table 3:** Relationship between disease activity and the development of carotid plaque in different CV SCORE risk categories.

	Carotid Plaque	
	OR (95% CI), *p*	
	Univariable	Multivariable
Low-risk SCORE category		
DAS28-PCR	**1.74 (1.12–2.71), 0.014**	1.57 (0.90–2.74), 0.11
Remission	-	-
Moderate activity	3.06 (0.89–10.52), 0.075	**5.00 (1.06–** **23.5), 0.042**
High and very-high activity	**4.38 (1.53–12.50), 0.006**	**4.18 (1.09–** **16.03), 0.037**
Moderate to high and very high-risk SCORE categories		
DAS28-PCR	1.10 (0.72–1.67), 0.68	1.05 (0.66–1.68), 0.83
Remission	-	-
Moderate activity	0.57 (0.16–2.04), 0.39	0.47 (0.12–1.83), 0.28
High and very-high activity	1.09 (0.35–3.44), 0.88	1.07 (0.33–3.60), 0.91

SCORE: Systematic Coronary Risk Evaluation. SCORE categories: Low (<1%), moderate (1–4%), high (5–9%) or very high (>10%). CV: Cardiovascular. DAS28: Disease Activity Score in 28 joints; CRP: C-reactive protein. Odds ratios (OR) are adjusted for age, diabetes, total cholesterol serum levels and the use of NSAIDs and prednisone Significant *p* values are depicted in bold.

## Data Availability

The data presented in this study are available on request to the corresponding author.
